# Improving pediatric idiopathic intracranial hypertension care: a retrospective cohort study

**DOI:** 10.1038/s41598-022-23960-w

**Published:** 2022-11-10

**Authors:** Adi Nitzan–Luques, Yarden Bulkowstein, Noa Barnoy, Adi Aran, Shimon Reif, Tal Gilboa

**Affiliations:** 1 Pediatric Department, Hadassah Medical Center, Ein-Kerem Campus, Jerusalem, Israel; 2 Faculty of Medicine, Hebrew University of Jerusalem, Jerusalem, Israel; 3 Pediatric Neurology Unit, Shaare Zedek Medical Center, Jerusalem, Israel; 4 Pediatric Neurology Unit, Hadassah Medical Center, Jerusalem, Israel; 5 Present Address: Pediatric Hemato-Oncology Department, Hadassah Medical Center, Ein-Kerem Campus, Jerusalem, Israel; 6 Present Address: Pediatric department, Schneider Children’s Medical Center of Israel, Petah Tikva, Israel

**Keywords:** Diseases, Health care, Neurology, Signs and symptoms

## Abstract

To describe the clinical course and prognosis of pediatric idiopathic intracranial hypertension (IIH) and examine the preferred management setting. IIH is characterized by increased intracranial pressure and is often associated with headaches and visual complaints. IIH is a preventable cause of vision loss in children. Hence, a rapid diagnosis followed by prompt treatment and follow-up is essential. However, standardization of the management of IIH in the pediatric population is not well established. Computerized medical charts of all 82 pediatric (< 18 years) patients diagnosed with IIH between 2007 and 2018 in the metropolitan area of Jerusalem were reviewed. Comparison was made between children followed in a multidisciplinary clinic in tertiary centers and those followed elsewhere. Detailed demographic and clinical data, as well as data regarding the follow-up setting and clinical course of the disease, were collected and analyzed. Recurrent IIH-related hospital returns were selected as a measurable marker for the uncontrolled disease. Recurrent IIH-related hospital return rate was significantly lower and occurred later among children followed by multidisciplinary teams compared to individual experts. Follow-up in multidisciplinary clinics improve the quality of life, and financial burden and may prevent permanent visual impairment in children with IIH.

## Introduction

Idiopathic intracranial hypertension (IIH) is a serious neurological disorder, which may be associated with frequent headaches, nausea, vomiting, vision loss, especially in peripheral vision, dizziness, neck stiffness, and even trouble walking. Without appropriate treatment, IIH may lead to blindness in up to 10% of patients^1^. Criteria for the diagnosis were modified several times and according to Friedman's criteria from 2013, includes now clinical, laboratory, and imaging characteristics (with or without papilledema)^2–4^.

The incidence of pediatric IIH was considered rare for many years. The estimated incidence in adults is 0.5–2 per 100,000 people per year in the general population and ten-fold higher in obese women of childbearing age^4,5^. The annual incidence in children might be slightly lower and is estimated at 0.5–1.2 per 100,000 people per year^6–8^. While pubertal pediatric patients have the same risk factors and female-to-male ratio as adults, prepubertal IIH patients show no gender predominance, with a lower obesity rate^9–15^. Moreover, worldwide, IIH is diagnosed at increasing rates in all ages, probably secondary to increased obesity rates and growing awareness^6,7,9,15,16^.

Little is known about the natural history of pediatric IIH. Unlike adults, children with IIH usually respond well and rapidly to medical treatment, with complete resolution of symptoms and papilledema^17,18^. Nonetheless, permanent visual impairment and relapse have been reported in up to 10–33% and 5–24% of pediatric patients, respectively^6,13,15,18–20^. High-grade papilledema and weight gain during the disease course have been established as predictors of poor visual outcome and a higher recurrence rate^19,21,22^. Contrariwise, the duration of symptoms before diagnosis, obesity at the time of diagnosis, and CSF opening pressure were found insignificant in most studies^1,13,18,23^. There are inconclusive findings regarding gender, age, and pubertal status affect prognosis.

The main objective of this study was to compare the short- and long-term outcomes of pediatric IIH patients followed in different clinical settings using unscheduled IIH-related emergency room (ER) visits and hospitalizations as markers of poor outcome. We hypothesized that multidisciplinary follow-up would improve patient care and decrease the need for unscheduled IIH-related hospitalizations and ER visits compared to follow-up by individual specialists.

The secondary outcome was to shed more light on the demographic and clinical characteristics of pediatric IIH patients, including the time to resolution of clinical symptoms and papilledema after medical treatment initiation, treatment modalities and duration and side effects of medical treatment.

## Subjects and methods

### Study design and population

This retrospective observational cohort study is based on multi-center computerized medical charts of pediatric patients clinically diagnosed with IIH between 2007 and 2018 in all three medical centers serving one metropolitan area (Jerusalem area) with a population of about 400,000 children. We performed a thorough computerized search using the terms: "intracranial hypertension", "pseudotumor cerebri,” "increased intracranial pressure,” "benign intracranial hypertension,” "headache,” "papilledema" and "diplopia.” We reviewed their medical records to detect children eventually diagnosed with IIH. This is the primary analysis of these data.

**Figure 1:**
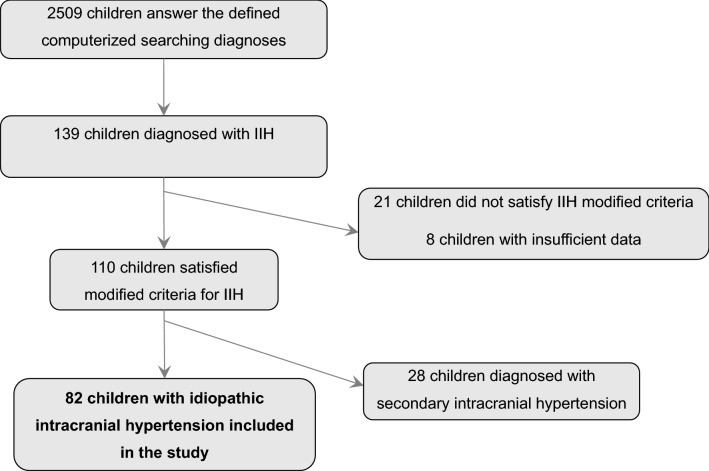
Study population selection flow chart. Inclusion and exclusion criteria.

The study population included pediatric patients under 18 years of age (at the time of diagnosis) diagnosed with IIH in one of the three participating medical centers. Inclusion in the study required fulfillment of the Friedman criteria for IIH^2,3^. Children who did not meet the criteria or had insufficient information to support the diagnosis were excluded. Note, that opening pressure measurements were carried out as part of the clinical inquiry and not as part of a study, therefore, were performed by various physicians. However, this test is done as customary under light sedation (using Ketamin/propofol) when the patient is in the lateral decubitus position with straight legs pre measurements. Children with a possible cause for the increased ICP (intracranial tumor, sinus venous thrombosis, intracranial hemorrhage, hydrocephalus, CNS infection, use of medication associated with increased ICP severe anemia, and other systemic disorders as detailed in Aylward and Reem review)^21^ were classified as secondary intracranial hypertension (SIH) and were excluded from this analysis (Fig. 1).

Notably, regardless of clinical presentation severity, all patients were instructed to continue medical follow-up by a multidisciplinary team in the medical center in which they were diagnosed. These multidisciplinary clinics provide a periodic joint examination by a board-certified pediatric neurologist and a board-certified pediatric ophthalmologist. All visits were carried out on the same day, in neighboring clinics, and clinical decisions were taken jointly by the neurologist and the ophthalmologist.

However, various factors such as economic considerations and parents' convenience made different types of medical sequels evident. Follow-up groups were classified as continuous follow-up by a multidisciplinary team in a tertiary center (follow-up group A) or follow-up by individual specialists in community clinics, not part of a multidisciplinary team (follow-up group B). Note, that our data lack information regarding patients who have been monitored in the community and have not made further visits to the emergency rooms.

### Study variables

Computerized medical charts were reviewed to collect detailed demographic and clinical data: age, gender, ethnic origin, weight and/or body mass index (BMI), medical history, clinical symptoms and physical examination at presentation, CSF opening pressure, and composition, laboratory, and neuroimaging studies. Due to a lack of documented Tanner stage, pubertal status was determined according to the patients' age. We defined patients as pubertal if they were over 10 years old for girls and 11 years old for boys at the time of diagnosis^24^. As height was not available for most patients who visited the ERs, BMI could not be calculated. Obesity was therefore defined as a body weight percentile ≥ 95% for age. Although we are aware of the limitations inherited in this imperfect parameter, using weight per age percentile as a nutritional state indicator is a common practice in ERs^25^. Moreover, there is a correlation between body weight percentile ≥ 95% and BMI ≥ 95% for age^26^. With the increasing rate of obesity in children, exceeding 20% in high-income countries, this correlation is likely to be substantial^27^. Data regarding medical management was collected as well, including treatment modalities, medication dosage, duration of treatment, and treatment side effects; clinical course including the time to resolution of symptoms, clinical recurrence, ER visits or hospitalizations due to IIH, duration, and type of follow-up (tertiary center follow-up versus another follow-up). We defined unscheduled ER visits or hospitalizations due to IIH as the primary outcome measure for poor outcome^28^. The frequency and prevalence for each variable were calculated for children with complete data. The institutional review boards approved the study.

### Statistical analysis

The study covered all hospitalizations in the Jerusalem area, thus minimizing selection bias. This is the primary data analysis, performed using IBM SPSS version 25 for Windows. The Pearson correlation coefficient was calculated to evaluate the strength of the association between two quantitative variables. Association between every two qualitative variables was tested using the Chi-square test or Fisher's exact test. Comparing quantitative variables between two categories of qualitative variables was performed using t-test analysis. The Kaplan–Meier survival model was used with the Log-rank test to compare survival curves for the evaluation of dichotomous outcomes depending on time from the initial presentation. The Cox regression model was applied to assess the effect of a quantitative variable on time-to-event of unscheduled hospital or ER admission. Sample size estimation was based on the expected difference in recurrent IIH-related hospitalizations between groups with a targeted significance level of *p*-value < 0.05 (two-tailed) and a power of 80%. All tests applied were two-tailed, and a *p*-value < 0.05 was considered statistically significant.

### Ethical approval

The study was approved by the Hadassah medical center Helsinki committee (HMO-0064-17) and was carried out according to the relevant guidelines and regulations. Informed consent was waived by the Hadassah medical center Helsinki committee since this is a retrospective analysis of de-identified data.

## Results

Using the computerized diagnoses mentioned above, we identified 2509 patients under 18 years of age who presented to one of the three medical centers in one metropolitan area between January 2007 and December 2018 with these diagnoses. After reviewing the charts, 110 were diagnosed with IIH. Of those 110 patients, 82 (74.5%) fulfilled the criteria for IIH and were included in the study (Fig. 1). The remaining 28 (25.5%) had a possible etiology or associated condition that may lead to raised ICP (SIH), and thus were excluded from the analysis.

### Demographic and clinical parameters (Table 1)

**Table 1:** Demographic and clinical characteristics at presentation of the study cohort.

	Follow-up Group A^a^	Follow-up Group B^b^	*p*-value
	n = 35 (42.7%)	n = 47 (57.3%)	
**Gender**			
Boys, n (%)	24 (68.6)	20 (42.6)	0.02
Girls, n (%)	11 (31.4)	27 (57.4)	
**Age (years)**			
Mean ± SD (range)	10.19 ± 3.78 (0.11–16.7)	11.31 ± 4.33 (1.2–17.5)	0.22
**Pubertal state ** ^**c**^			
Prepuberal, n (%)	19 (54.3)	20 (42.6)	0.29
Pubertal**,** n (%)	16 (45.7)	27 (57.4)	
**Ethnic origin**			
Jewish, n (%)	27 (77.2)	33 (70.2)	0.48
Arab, n (%)	8 (22.8)	14 (29.8)	
**Obesity** ^**d**^			
n (%)	18 (51.4)	19 (40.4)	0.32
**Symptoms**			
Headache, n (%)	28 (80)	39 (83)	0.73
Visual disturbance, n (%)	17 (48.5)	32 (68.1)	0.07
Nausea/ vomiting, n (%)	8 (22.8)	12 (25.5)	0.78
Tinnitus, n (%)	2 (5.7)	1 (2.1)	0.39
Asymptomatic, n (%)	2 (5.7)	2 (4.2)	0.76
**Clinical signs**			
CN VI palsy, n (%)	6 (17.1)	15 (32)	0.12
Papilledema, n (%)	32 (91.4)	38 (80.8)	0.18
CSF opening pressure, mean ± SD (range), cm of water	39.7 ± 9.2 (28–60)	39 ± 7.9 (28–55)	0.74

SD, standard deviation; CN, cranial nerve; NA not available.

^a^Continuous follow-up in tertiary center; ^b^Follow-up in community clinics or no follow-up at all; ^c^Girls over the age of 10 years and boys over the age of 11 years; ^d^Weight percentile ≥ 95%, data was not available for 5 children.

Demographic parameters of the study groups, such as gender, age, puberty status, ethnic origin, and obesity, are presented in Table 1. The average age was 10.83 ± 4.12 years; girls were the majority (67%) in the pubertal age group (Supp. Table 1), and 65% of the pubertal age group were obese (weight > 95% percentile). The research population included all ethnic origins, at the same ratio as expected by their known proportion in the population^29^. Group A had twice as many boys as girls, while in group B, there were slightly more girls. There was no difference in age, puberty status, ethnic origin, or obesity rate between the groups.

Clinical symptoms and signs of the patients in the two follow-up groups are detailed in Table 1. The most common symptom was a headache, followed by visual disturbance, nausea, vomiting, and tinnitus. In each follow-up group, two children were asymptomatic and were diagnosed after an incidental discovery of papilledema during a routine ophthalmological exam. Papilledema was detected in 91.4% and 80.8% of patients in follow-up groups A and B, respectively. The CSF average and median opening pressure was similar in both follow-up groups. Complete demographic and clinical data were available for all participants.

Demographic characteristics of prepubertal and pubertal groups are described in Supp. Table 1. As expected, there was a significant female and obesity predominance in the pubertal group with IIH. In contrast, in the prepubertal group, there was a lower rate of obesity and male predominance (Supp. Table 1).

### Treatment

All children in follow-up group A and most of the children (93.6%) in follow-up group B were initially treated with acetazolamide. Additional second-line treatments included steroids (8.6%), furosemide (2.8%), and topiramate (11.4%). Data regarding acetazolamide treatment was available for 29 (82.8%) children in follow-up group A and 15 (32%) children in group B. In follow-up group A, the mean duration of acetazolamide treatment was 7.8 ± 4.67 months; The maximal dosage ranged from 5.5 to 55 mg/kg/d with a mean of 20.06 ± 9.99 mg/kg/d; Clinical adverse effects were reported in 12 (34.2%) children including neurologic side effects in 22% (mainly paresthesia and fatigue), GI side effects in 20% (mainly diarrhea and nausea and vomiting) and acute renal failure in one child (led to a change of medication); Metabolic acidosis during the treatment period was documented in 13 (37.1%) patients. However, the information regarding the treatment duration, medication dosage, side effects or metabolic status in most patients in follow-up group B was partial, and thus was not analyzed.

Though this did not reach statistical significance, the rate of repeat LPs was higher in follow-up group B: eleven out of 47 in group B (23%) compared to three out of 35 in follow-up group A (8.6%) (*p* = 0.077). Finally, only one patient in follow-up group A required a neurosurgical procedure due to a persistent, refractory IIH, compared to three children in follow-up group B (Table 2).

**Table 2:** Primary outcome of the follow-up groups.

	Follow-up Group A^a^	Follow-up Group B^b^	*p*-value
	n = 35 (42.7%)	n = 47 (57.3%)	
**Patients with unscheduled IIH-related hospital visits, n (%)**	5 (14.3)	20 (42.5)	< 0.006
1 visit, n (%)	3 (8.6)	12 (25.5)	
2 visits, n (%)	1 (2.8)	5 (10.6)	
3 visits, n (%)	1 (2.8)	3 (6.3)	
Patient with prolonged unscheduled admission ^c^, n (%)	0 (0)	8 ^d^ (17)	< 0.05
Mean unscheduled hospital stay per patient, days	0.37	1.85	< 0.03
Repeated lumbar punctures, n (%)	3 (8.6)	11 (23)	0.08
Neurosurgical treatment, n (%)	1 (2.8)	3 (6.4)	0.54

SD, standard deviation; ^a^ Continuous follow-up in tertiary center; ^b^ Follow-up in community clinics or no follow-up at all; ^c^ hospital stay longer than 96 h; ^d^ One patient had two prolonged admissions.

### Follow-up and clinical course

Though all study participants were instructed to continue medical follow-up by a multidisciplinary team in a tertiary center clinic, more than half did not comply with the recommendation. Follow-up groups A and B included 42.7% and 57.3% of the study participants, respectively. As mentioned above, described here is data collected over 12 years. The mean follow-up duration in group A was 21.67 ± 21.81 months (from 1.5 months to 7.4 years) and was unknown for group B.

The mean time to clinical resolution in group A was 2.22 ± 2.84 months (median 1 month, Q1 0.5, Q2 1, Q3 2.75, range 0.1–12 months). Clinical resolution (i.e., resolution of headache and visual complaints) usually preceded the papilledema resolution, which was documented after the mean of 7.8 ± 8.9 months in group A (median 5.25 months, Q1 2.65. Q2 5.25, Q3 8.5, range 0.25–41 months). This data was unavailable for most, group B patients. However, the data from follow-up group A sheds light on the natural course and prognosis of children with IIH under optimal care in multidisciplinary clinics in tertiary centers.

**Figure 2:**
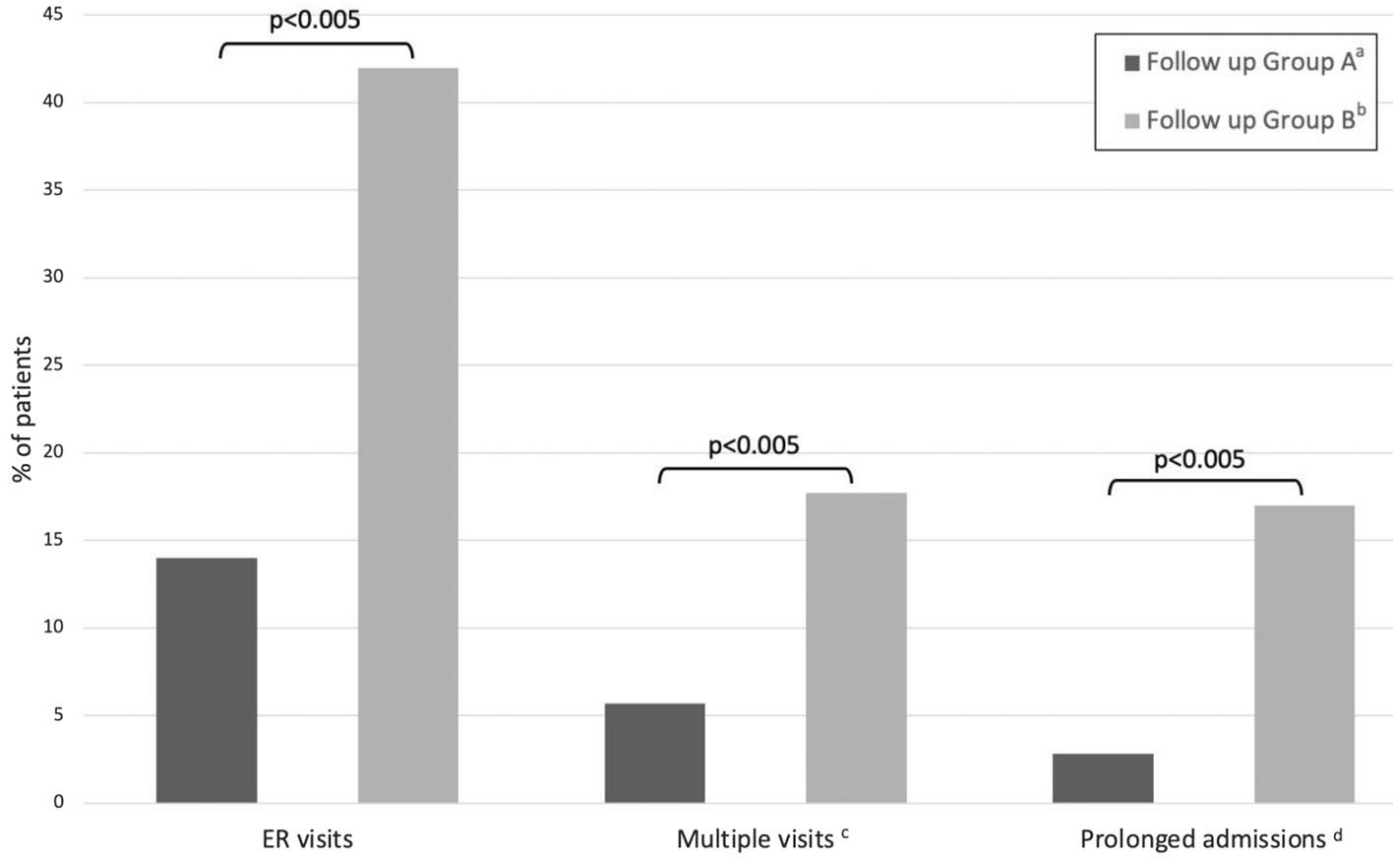
Study primary endpoints. IIH-related ER visits and hospitalization days among the different follow-up groups. Children followed in a tertiary center multidisciplinary clinic (follow-up group A) had a significantly lower rate of unscheduled IIH-related ER visits, multiple ER visits and prolonged IIH-related admissions (*p* < 0.005). ^a^Continuous follow-up in tertiary center; ^b^Follow-up in community clinics or no follow-up at all; ^c^> 1 ER visits; ^d^> 96 h hospital stay.

**Figure 3:**
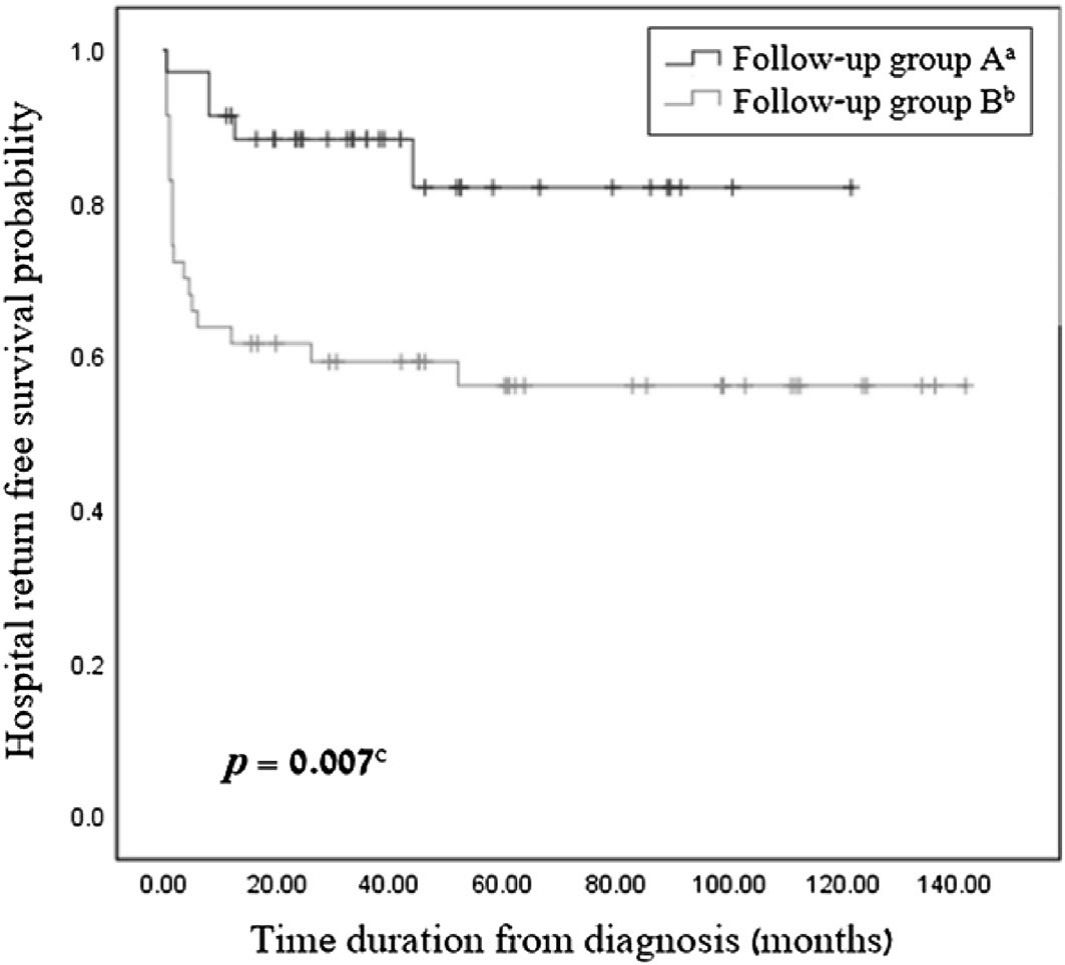
Incidence of IIH-related first unscheduled hospital visit in pediatric population. Kaplan–Meier survival analysis describes the incidence of IIH-related unscheduled hospital return of children diagnosed with IIH that were followed up continuously in tertiary center (follow-up group A) and children with another follow-up or no follow-up at all (follow-up group B). ^a^Continuous follow-up in tertiary center; ^b^Follow-up in community clinics or no follow-up at all; ^c^Log-rank test.

Figure 2 presents the primary endpoints of the study. Children followed in a tertiary center multidisciplinary clinic (follow-up group A) had a significantly lower rate of unscheduled IIH-related ER visits (*p* < 0.005), a lower rate of multiple ER visits (*p* < 0.005), and a lower rate of prolonged IIH-related admissions (*p* < 0.005). Table 2 describes the outcome of the follow-up groups. Children in follow-up group A had a mean of 0.22 unscheduled IIH-related ER visits per patient, compared to 0.65 in group B. The odds ratio for an unscheduled ER visit in group A compared to group B was 0.225 (95% CI 0.07, 0.68). Detailed data regarding number of visits in each group are presented in Table 2. Furthermore, the Kaplan–Meier survival analysis (Fig. 3) shows a significantly longer time from diagnosis to the first unscheduled ER visit in follow-up group A compared to group B (*p* = 0.007). Regarding unscheduled hospital admissions, the mean stay was one day of hospitalization per patient in group A, compared to 1.85 days per patient in group B (Table 2).

The time and reason for returning to the hospital are presented in Supp. Figure 1. The first return occurred on average 7.83 months (median 1.5 months) after diagnosis. The most common reason for the first return was treatment non-responsiveness (64%), followed by recurrence after stopping treatment (28%) and treatment side effects (8%).

The type of follow-up was the only significant factor associated with recurrent unscheduled ER and hospital admissions. Age, gender, ethnic origin, puberty state, obesity, and opening pressure were similar in those who returned to the hospital and those who did not.

## Discussion

IIH is a serious disorder with significant implications that may eventually cause permanent visual impairment along with additional characteristic clinical sequela as ultimately described above. The natural history and clinical course of pediatric IIH are not well established, and different predicting factors for the clinical course and prognosis have been investigated. Though the condition has been known for over 80 years, data regarding the quality of the management of pediatric IIH is missing, and there is no standardization of follow-up and treatment protocols^30–32^.

This multicenter study describes the clinical presentation, disease course, and prognosis of children diagnosed with IIH and investigates the correlation between the type of follow-up and the long-term disease course and outcome, manifested as unscheduled IIH-related hospital returns (ER visits and re-hospitalizations).

Our cohort is comparable to other cohorts of pediatric IIH patients regarding age, gender distribution, demographic features, clinical presentation, and disease course^21,33^. Similar to other studies, we noted a higher prevalence of boys and a lower rate of obesity in the prepubertal group, along with female and obesity predominance in the pubertal group^6,11,15,19,34,35^. Follow-up group A consisted of a higher rate of boys, possibly explained by the younger age of male patients in the cohort. Clinical features, including LP opening pressure, were similar among the gender groups. Accordingly, boys in our cohort seem to have a favorable outcome, though the difference is not significant. Nonetheless, this variance may result from the relatively small group size. However, in our study, age, gender, ethnic origin, puberty state, obesity, and CSF opening pressure at diagnosis did not influence the prognosis.

We did not find a correlation between pubertal state (as well as older age) and worse prognosis, as reported by Stiebel-Kalish et al.^36^. Different outcome measures may explain this difference. We used unscheduled admission as a marker of uncontrolled IIH, and in Stiebel-Kalish’s study, by the visual outcome. Similar to Sheldon et al. et al. findings, obesity was more prevalent in pubertal patients in our study^33^. However, neither age nor obesity were associated with poor prognosis in our study. It is possible that our groups were too small, and the type of follow-up was significantly more in determining the outcome than any other factor.

It is important to note that the clinical definition of “poor outcome” may not align with patient experience. A future study that incorporates a measure of patient-reported disability (analogous to PedMIDAS score in migraine populations) should be carried out.

Time to clinical resolution (i.e., resolution of headache and visual complaints) in our study was, on average, 2.22 ± 2.84 months. This is similar to the reported time to resolution by others.^6,21,23^ Papilledema resolution was documented after the mean of 7.8 ± 8.9 months in the multidisciplinary tertiary center follow-up group. This data was not available for most of the group B patients. Time to papilledema resolution in other studies varied between 4.2 and 5 months^21^. This difference may be explained by more frequent visits, treatment protocols, or different definitions of papilledema resolution.

Factors affecting CSF opening pressure in children have been investigated, with inconsistent findings through different studies. Higher CSF opening pressure was found in correlation with older age and obesity^36–40^. This study found that a higher weight percentile was weakly correlated with higher CSF opening pressure. However, consistent with other reports, no correlation was found between gender or age and CSF opening pressure^10,19,21,37,39^.

In agreement with our initial hypothesis, we found that children who were under the care of a multidisciplinary team in a tertiary center had significantly lower odds of returning to the hospital, a lower rate of resulting inpatient days, as well as longer time intervals between hospital returns, compared to children followed by individual specialists elsewhere. Furthermore, though not statistically significant, the rate of repeat LPs was lower in the group with multidisciplinary follow-up.

Although not specifically evident from the data collected within the frame of this work, it is most probable that non-concordance with follow-up (i.e. group B) reflects as well a general non-concordance manner, including adherence to medication plans. This in itself may carry with it adverse effect outcomes and eventually lead to “poor outcome “, which was associated in this work mainly with the lowest quality of follow-up.

Indeed, several publications have suggested that before, but to the best of our knowledge, this is the first study that confirms that assumption^21,41,42^. This finding is likely related to several advantages of tertiary center clinics, where pediatricians, neurologists, ophthalmologists, and other IIH-related specialists work in concert. This multidisciplinary approach improves the outcome of children with IIH compared to follow-ups by specialists in other settings. Moreover, greater availability of diagnostic tools, better collaboration between the different specialists taking care of the patient, no need for making separate appointments with different doctors, and immediate access to laboratory and imaging studies lead to faster management adjustments if needed.

Several other explanations for this identified difference between follow-up groups might be that group B contained more private practice providers who recommended ER visits because they were unable to accommodate them in the clinic. Alternatively, private practice providers may teach patients to go first to ER for issues. Unfortunately, we do not have data on unplanned clinic visits that may have been an alternative to a visit in the ED. There is a place for additional, comprehensive prospective future study.

IIH-related hospital returns in our study were usually derived from continuation or relapse of IIH symptoms as well as treatment-related adverse events. We assume that treatment failure is associated with poor outcomes and will likely lead to ER visits and hospitalizations due to the continuation or recurrence of symptoms. In addition to improved prognosis, appropriate care prevents other aspects of ER visits and hospitalization. They are associated with significant impairment of quality of life for the child and his family, risk of infection, and financial expenses to the health system^43,44^. Thus, minimizing these events is crucial and may be achieved using appropriate long-term management.

A recent study estimated the financial burden of pediatric hospitalization as 2,192 USD (mean individual medical cost) for the entire hospitalization period^45^. In Israel, the direct cost of a pediatric ER visit and inpatient hospitalization day are 280 and 1000 USD, respectively (charges defined by the Israeli national health system (NHS))^46^. Additional effects include loss of income and non-medical expenses, estimated at a median of 51 USD per day. The economic impact is more significant in lower socioeconomic populations than in others^47^. Based on this data, the multidisciplinary tertiary center follow-up in our study (follow-up group A) saved the NHS about 58440 USD (23 ER visits and 52 inpatient days), as well as considerable non-medical expenses, significantly among families in the low socioeconomic status. In follow-up groups A and B, the direct medical expenses due to ER visits and re-hospitalizations were 1000 and 1900 USD per inpatient day, respectively. Of note, this difference is likely to be even more significant since most hospitalization days in group A were due to a single patient.

IIH-related hospital returns in our study mainly occurred during the first year after diagnosis. Hence, follow-up evaluations should be more frequent during the first year of the disease course to prevent recurrent hospital returns, with longer intervals in the following years. However, some occurred several years later (up to 5.4 years after the diagnosis), emphasizing the importance of long-term follow-up.

The main strength of this study is its multi-center scope, detecting pediatric IIH cases from all three medical centers in Jerusalem and the surrounding area, and providing medical care for more than 400,000 children. As mentioned earlier, selection bias was minimized by covering all hospitalizations in the Jerusalem area.

One of the study’s significant limitations is its retrospective nature and the dependence of data collection on the quality of computerized documentation. Thus, we reluctantly used weight percentiles to define obesity rather than BMI because height is not routinely recorded in the pediatric emergency department. Age was used to determine puberty since the Tanner stage is not routinely documented. Furthermore, data on pediatric patients who did not have a follow-up in tertiary centers (follow-up group B) was limited. It was not possible to fully characterize their management and the course of their disease.

A gender imbalance characterizes our two compared research groups as described above. This may serve as s a potential confounder. A future large-scale study may eliminate this bias and shed light on the contribution of gender to follow-up adherence and treatment compliance.

Finally, we had only partial information about ophthalmological assessment, such as the visual field and optic nerve function. Hence, further prospective studies are desired and may add information on the ophthalmological natural course and management-related outcomes.

Based on the results of this work, we assigned a designated clinic coordinator, who is in charge of scheduling appointments for newly diagnosed patients with IIH prior to their hospital discharge and helping with any future challenges in attending clinic appointments. Moreover, the same pediatric neurologist and pediatric ophthalmologist follow the patients long-term, and the clinic visit is now executed jointly in the same room. A future study to assess the outcomes after the implantation of the described improvements is warranted.

In conclusion, this study provides new insight into the clinical course and management of pediatric IIH patients. Poor disease control may lead to unscheduled IIH-related hospital visits that may occur shortly after the initial diagnosis or even years later. Decreased rates of recurrent IIH-related hospital returns and hospitalization days and longer time to hospital returns were related to multidisciplinary follow-up in tertiary center clinics compared to follow-up in community clinics.

This retrospective study has generalizability (external validity) quality since it surveyed various parameters of a diverse, heterogeneous population of subjects (age, gender, ethnicity, etc.) comparably in both study groups. Based on these findings, this study provides insight into how hospital/medical systems can design care models to serve patients and avoid high healthcare costs associated with emergency room/inpatient visits. Long-term, coordinated follow-up by a multidisciplinary team in a tertiary center probably improves pediatric IIH patients' life quality, decreases hospital-acquired infections, saves hospitalizations-induced expenses, and possibly prevents permanent visual impairment.

## Supplementary Information

Supplementary Information.

## Data Availability

The anonymized data is available for review upon request. Researchers may contact the corresponding author.

## Abbreviations

IIH: Idiopathic intracranial hypertension
CSF: Cerebrospinal fluid
ER: Emergency room
BMI: Body mass index
ICP: Increased intracranial pressure
