# Supplementary material for: Improving pediatric idiopathic intracranial hypertension care: a retrospective cohort study
Source: Sci Rep. 2022 Nov 10;12:19218. doi: 10.1038/s41598-022-23960-w (PMC9649632; doi:10.1038/s41598-022-23960-w)
Supplement: Supplementary file 1 — Supplementary Information. [file 41598_2022_23960_MOESM1_ESM.docx]

**SUPPLEMENTAL**

**Supp. Figure 1** Time and reason for IIH-related hospital returns


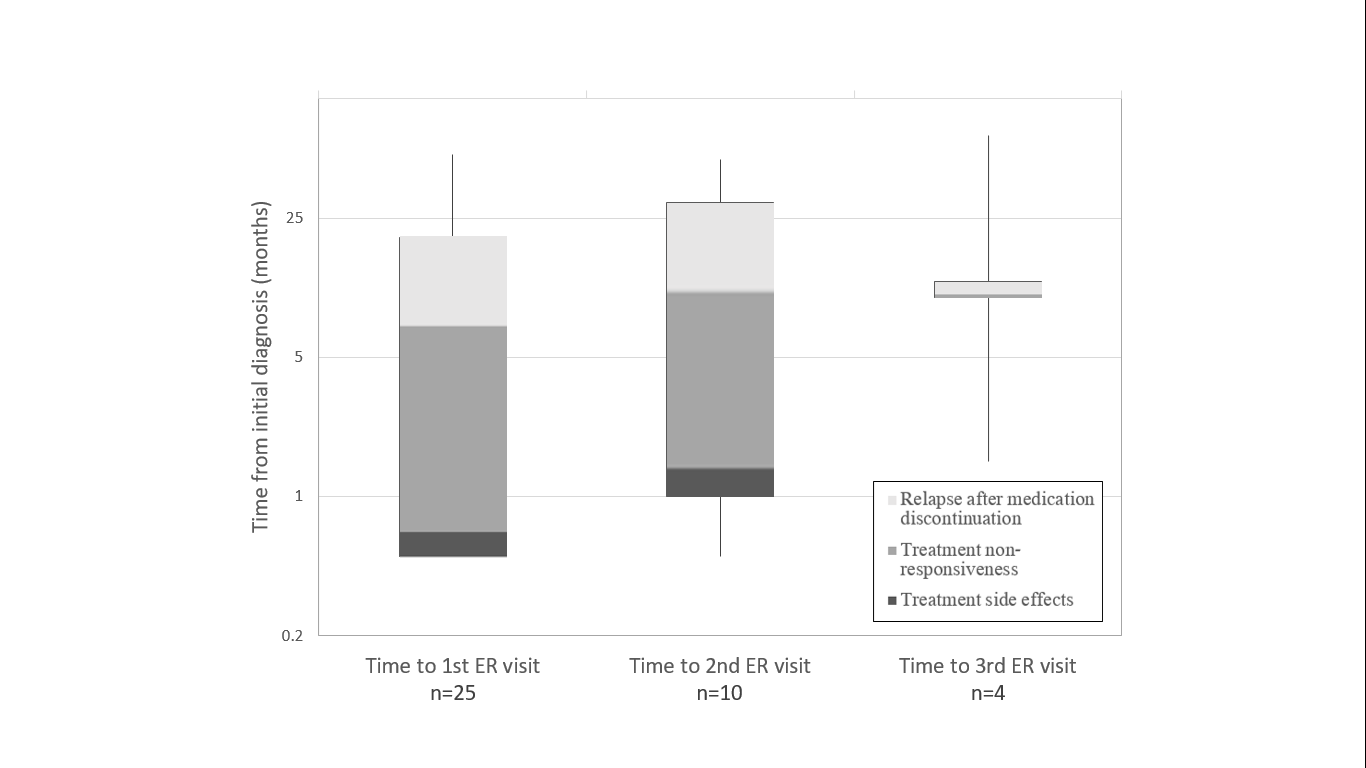


Time to IIH-related ER visits after the initial diagnosis on the logarithmic time scale, the boxes range <represent the mean time to visit ± 1 SD. Each box is divided according to the reason for the ER visit, as detailed.

**Supp. Table 1** Demographic characteristics of prepubertal and pubertal groups with IIH

|  | **Prepubertal**  (n=39) | **Pubertal^a^**  (n=43) | ***p*-value** |
| --- | --- | --- | --- |
| **Gender** |  |  | **<0.001**^b^ |
| Boys, n (%) | 30 (76.9%) | 14 (32.6%) |  |
| Girls, n (%) | 9 (23.1%) | 29 (67.4%) |  |
| **Age** |  |  |  |
| Mean ± SD, years | 7.39 ± 2.95 | 13.95 ± 2 |  |
| Range, years | 0.11-10.9 | 10.11-17.5 |  |
| **Ethnic origin** |  |  | 0.465^b^ |
| Jewish, n (%) | 30 (76.9%) | 30 (69.8%) |  |
| Arab, n (%) | 9 (23.1%) | 13 (30.2%) |  |
| **Obesity** |  |  | **0.002**^b^ |
| Obese^c^, n (%) | 11 (29.7%) | 26 (65%) |  |

IIH = Idiopathic intracranial hypertension

^a^ Girls over the age of 10 years and boys over the age of 11 years

^b^ Chi-square test

^c^ Weight percentile ≥ 95%, data was not available for five children
